# Filtering failure: the impact of automated indexing in Medline on retrieval of human studies for knowledge synthesis

**DOI:** 10.5195/jmla.2025.1972

**Published:** 2025-01-14

**Authors:** Nicole Askin, Tyler Ostapyk, Carla Epp

**Affiliations:** 1 Nicole.askin@umanitoba.ca, University of Manitoba, Manitoba, Canada; 2 tyler.ostapyk@umanitoba.ca, University of Manitoba, Manitoba, Canada; 3 Carla.epp@umanitoba.ca, University of Manitoba, Manitoba, Canada

**Keywords:** Evidence Synthesis, Abstract and Indexing, Medical Subject Headings (MeSH), Automated Indexing

## Abstract

**Objective:**

Use of the search filter ‘exp animals/not humans.sh’ is a well-established method in evidence synthesis to exclude non-human studies. However, the shift to automated indexing of Medline records has raised concerns about the use of subject-heading-based search techniques. We sought to determine how often this string inappropriately excludes human studies among automated as compared to manually indexed records in Ovid Medline.

**Methods:**

We searched Ovid Medline for studies published in 2021 and 2022 using the Cochrane Highly Sensitive Search Strategy for randomized trials. We identified all results excluded by the non-human-studies filter. Records were divided into sets based on indexing method: automated, curated, or manual. Each set was screened to identify human studies.

**Results:**

Human studies were incorrectly excluded in all three conditions, but automated indexing inappropriately excluded human studies at nearly double the rate as manual indexing. In looking specifically at human clinical randomized controlled trials (RCTs), the rate of inappropriate exclusion of automated-indexing records was seven times that of manually-indexed records.

**Conclusions:**

Given our findings, searchers are advised to carefully review the effect of the ‘exp animals/not humans.sh’ search filter on their search results, pending improvements to the automated indexing process.

## INTRODUCTION

Knowledge synthesis searching attempts to comprehensively retrieve all published literature on a particular question using a replicable search strategy. To address the volume of literature retrieved for broad or popular topics, information specialists often incorporate search filters to focus the search to particular types of records. Many of these filters designed for the Medline or PubMed databases use Medical Subject Heading (MeSH) controlled vocabulary terms. One particularly common strategy is the use of “double NOT” or exclusion filters to rapidly exclude irrelevant results based on subject headings. The Cochrane Highly Sensitive Search Strategies use the string ‘exp animals/not humans.sh’ combined with NOT against the rest of the search strategy to limit searches to human studies [1]. Variants of this string have been widely adopted as standalone filters [2, 3] and as parts of other filters [4, 5].

The National Library of Medicine (NLM) implemented fully automated indexing of Medline in a graduated process, beginning with a pilot of 8 journals in 2019, 40% of journals in 2021, and 100% of journals by April 2022. Their method used the Medical Text Indexer-Automatic (MTIA), a natural language processing-based system, to assign MeSH terms [6]. MTIA identifies MeSH terms, synonyms, and trigger phrases in the title and abstract of records and incorporates position and frequency analysis in determining how to index an article [7]. In announcing the automated indexing transition, NLM highlighted improved timeliness and ability to scale indexing to meet the expanding volume of published literature as key drivers underlying the initiative [8]. However, anecdotal reports circulated among medical librarians and information specialists about failings in MeSH terms applied by the automated method, exemplified by the social media hashtag #meshfail. This raised questions about the continued reliability of MeSH-based searches and search filters.

While there is limited extant literature on the impact of automated indexing in Medline on information specialist practice, what does exist seems to bear out these concerns. For example, Hickner [9] conducted interviews with systematic searchers regarding search systems in which a respondent noted a concern about the impact of automated indexing on search precision. Chen and colleagues reported frequently missing or misused check tags, along with an apparent gender bias in ranking the Male/heading over Female/[10]. Koning and colleagues applied the MTI algorithm to texts from patent applications, and found that for the application of the Female/subject heading the algorithm had a precision of 93% but recall of only 65% [11]. Most significantly, the work of Amar-Zifkin and colleagues identified multiple concerns with automated indexing in Medline, including irrelevant terms being included, obviously relevant terms being absent, and cases where better terms were available but unused [12, 13]. Causes they noted for these issues included misinterpretation of acronyms, rhetorical or metaphorical language, words that occur in multiple MeSH terms, and “unusual combinations of populations-interventions.” They reported that nearly half of the records they examined exhibited one or more inadequacies.

In this study we sought to understand the impact of the switch to automated indexing on the use of purely MeSH-based filtering for knowledge synthesis. Specifically, we examined the use of the common exclusion filter ‘exp animals/not humans.sh’, part of the Cochrane Highly Sensitive Search Strategies for identifying randomized trials [1]. We separated studies by indexing method—whether automated, curated or manual—and compared the frequency with which human studies were incorrectly excluded by this filter.

## METHODS

**Figure 1:**
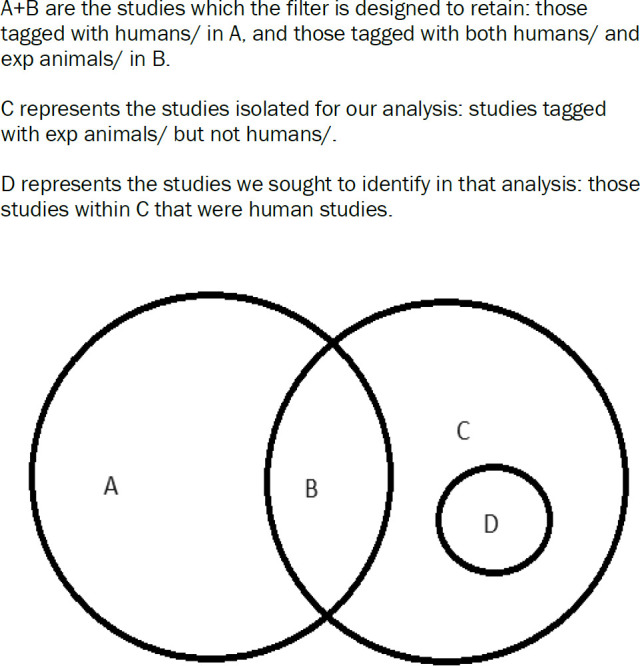
Venn diagram of Boolean logic model.

We searched Ovid Medline on 10 March 2023 using the Cochrane Highly Sensitive Search Strategy for identifying randomized trials in Medline, sensitivity- and precision-maximizing version, 2008 revision, Ovid format [1]. Results specifically excluded by the filter ‘exp animals/not humans.sh’ were isolated and limited to Medline records with a publication year of 2021 and 2022 to capture a sample of records spanning the transition to fully automated indexing. Figure 1 shows the logic model for the Boolean used.

These results were then divided into sets based on the indexing method field (.ig). According to the Ovid database guide [14], there are three values that can be determined using this field:

- “Automated” reflects a record for which MeSH indexing is provided algorithmically
- “Curated” indicates that “MeSH indexing is provided algorithmically and a human reviewed (and possibly modified) the algorithm results”
- “Manual” is the designation we used for cases in which the field was not present, meaning “the indexing method is fully human indexed” [14].

This approach produced 4865 results for the automated set, 3062 for the curated set, and 2517 for the manual set. The full search strategy is available in Appendix A.

Each set was uploaded to a separate review in the knowledge synthesis platform Covidence, which automatically removed duplicates. The results were then screened blindly by two reviewers per record at both the title-abstract and full-text stages.

Records were included if they described a human study; no other study-type restrictions were imposed at the screening stage. For the purposes of this project, a very broad definition of human study was used, encompassing in vitro and ex vivo studies. Studies involving both humans and animals were considered human studies if one of the following cases applied: (1) the study was interventional and the intervention was performed on a human or human product (human cells, tissues, etc.); (2) the study was comparative and involved a comparison directly between groups of humans; or (3) in any study type, a significant outcome of interest was focused on humans. Studies involving human products administered to animal subjects were excluded unless the human product was cellular and was manipulated or analyzed in some way prior to administration. If a single paper described multiple studies, it was included if any of those studies met the definition of human study in use. For full-text screening of non-English studies, the Deepl translator was used to facilitate evaluation. The following exclusion rationales were applied in full-text screening:

- Animal study: the study is entirely on animals or animal products (animal cells, tissues, milk, etc.) and involves no human subjects or human products
- Wrong study type: the study does not have either human or animal subjects
- Ag/vet: the study is agricultural or veterinary in nature and does not meet any of the inclusion criteria to be considered human
- Human product: the study involved administration of an unmanipulated/unanalyzed human product to animals
- Unable to obtain full text through library subscriptions or interlibrary loan

PRISMA flow diagrams [15] for all three sets are included in Appendix B.

We created and pre-tested a custom data extraction form for analysis of the included studies. In designing the data extraction form we sought to include factors that may impact accuracy of indexing, including the presence of an abstract, the language of the full study, and whether the paper described multiple studies. We provided a list of study designs and study contexts (clinical, preclinical, educational, agricultural, veterinary, and other) with definitions to improve selection accuracy. We also extracted data related to the population, intervention, and indexing characteristics of the records. Finally, the form included an optional free-text field in which the extractor could propose potential reasons for the study to have been misinterpreted as an animal study. We used a two-reviewer model for the extraction process, with one reviewer serving as data extractor and the second as data verifier.

## RESULTS

Each set of results included human studies based on the criteria set out in this project. We identified 205 human studies out of 4865 in the automated indexing set, 69 human studies out of 3062 in the curated indexing set, and 56 human studies out of 2517 in the manual indexing set. Thus, 4.2% of the articles in the automated indexing set were found to be human studies, compared to 2.3% in the curated set and 2.2% in the manual set.

We considered that the language of publication of the full-text study might impact the accuracy of the automated indexing compared to manual indexing, which includes access to full text. However, the manual set contained no non-English studies, so we were unable to assess the impact of language on this indexing method. The automated set had 522 non-English studies, 15 of which (2.9%) were classified as human studies, and the curated set had 238 non-English studies, 3 of which (1.3%) were classified as human studies.

Many of the human studies records described multiple studies: 53 out of 205 (25.9%) in the automated set, 35 out of 69 (50.7%) in the curated set, and 23 out of 56 (41.1%) in the manual set. For example, a single record might describe an animal RCT as well as a human epidemiologic study. Records in this category may have been appropriately indexed as animal studies but missed the addition of the humans/check tag. Similarly, there were some review articles which included analysis of both human and animal primary literature that were incorrectly indexed as solely animal studies. We conducted an analysis of the study type(s) considered a human study according to our definition. The distribution is shown in Table 1, although some multi-study records describe multiple human studies.

**Table 1:** Human studies by study type per set.

Type	Automated	Curated	Manual
RCT	85	14	17
NRE	44	29	16
Epidemiologic	20	12	3
Review	43	9	10
Qualitative	2	2	3
Case series/report	2	0	0
DTA	7	1	1
Economic	2	0	2
Opinion	1	0	0
Other	7	3	5

We additionally categorized included records according to study context: clinical, preclinical (laboratory), educational, and other. Because of our expansive definition of human studies, some records with an agricultural or veterinary context were also eligible for inclusion. As with study type, some records described multiple study contexts, although this was less frequently an issue. The distribution is presented in Table 2.

**Table 2:** Distribution of human studies by study context per set.

Context	Automated	Curated	Manual
Clinical	134	27	17
Preclinical	102	37	32
Educational	4	1	0
Agricultural	9	3	4
Veterinary	6	1	3
Other	4	2	1

Combining these two datasets allows us to determine the incorrect exclusion of human studies by type and context for each indexing method. We conducted a subanalysis on the clinical subset since this is the context in which the Cochrane RCT filter was developed. The exclusions by study type in the clinical context are displayed in Table 3.

**Table 3:** Exclusions of clinical-context records by study type and indexing method. Percentage of total set represents the entire set of articles excluded by the filter ‘exp animals/not humans.sh’ for that indexing method.

	Automated		Curated		Manual	
	Count	% of total set (n=48 65)	Count	% of total set (n=30 62)	Count	% of total set (n=25 17)
RCT	67	1.38%	7	0.23%	5	0.20%
NRE	7	0.14%	4	0.13%	0	0.00%
Epidemiolo gic	14	0.29%	7	0.23%	0	0.00%
Review	40	0.82%	8	0.26%	10	0.40%
Qualitative	1	0.02%	0	0.00%	0	0.00%
Case	1	0.02%	0	0.00%	0	0.00%
DTA	4	0.08%	1	0.03%	0	0.00%
Economic	0	0.00%	0	0.00%	2	0.08%
Opinion	1	0.02%	0	0.00%	0	0.00%
Other	1	0.02%	1	0.03%	1	0.04%

In addition to the quantitative analysis, we conducted a qualitative review of the record abstracts to see whether we could discern the reasons for which records may have been incorrectly indexed. The reasons are summarized in Table 4.

**Table 4:** Potential reasons for animal indexing, by indexing method.

	Automated		Curated		Manual	
	Count	% of included records (n=205)	Count	% of included records (n=69)	Count	% of included records (n=56)
Unknown	3	1.46%	1	1.45%	0	0.00%
Agricultural or veterinary context	16	7.80%	5	7.25%	8	11.59%
Allergy-related study	3	1.46%	0	0.00%	0	0.00%
Animal product (meat, milk, etc.)	39	19.02%	4	5.80%	2	2.90%
Animal model (e.g. tor evaluating a surgical procedure)	5	2.44%	3	4.35%	3	4.35%
Human product administere d to an animal population	9	4.39%	3	4.35%	8	11.59%
Mentions excluding animals	3	1.46%	0	0.00%	0	0.00%
Includes animal study	87	42.44%	43	62.32%	28	40.58%
Animal-related language (e.g. “click a mouse”)	5	2.44%	2	2.90%	2	2.90%
Mentions prior animal work	32	15.61%	4	5.80%	0	0.00%
Animal-borne/zoon otic diseases	5	2.44%	4	5.80%	8	11.59%
Pet-related study	5	2.44%	0	0.00%	0	0.00%
Other	3	1.46%	2	2.90%	0	0.00%

The most common reason for inappropriate indexing across all sets was that the record included animal studies – whether because the record described multiple studies, or for example, a review or opinion paper discussed both human and animal work. The comparatively high proportion of agricultural, veterinary, animal-disease, and human-product studies in the manually curated set is likely related to our particular definition of human studies.

However, some of the reasons for inappropriate indexing specific to the automated indexing set warrant further evaluation. Of the 39 studies that may have been excluded due to inclusion of an animal product, 17 (44%) involved a dietary intervention, suggesting that this topic of research may be significantly impacted by inappropriate indexing. Other animal-product exclusions concerned use of animal tissue in transplantation or in the development of vaccines.

## DISCUSSION

Our analysis demonstrated that human clinical RCTs are excluded by the ‘exp animals/not humans.sh’ filter in automated-indexing records at six times the rate of curated-indexing records and nearly seven times the rate of manually-indexed records. Concerningly, the mention of prior animal work in the abstract was a very common reason for inappropriate indexing, as the algorithm is unable to understand that this mention is not what the article is “about”. Along those same lines, some records were indexed as animal studies when their abstracts specifically mentioned excluding animals (particularly in reviews). Finally, studies which included an animal-related intervention (such as a pet) or problem (such as an allergy) were indexed using terms related to the animal(s) involved but had no corresponding humans/check tag.

Given our findings, we urge information specialists conducting knowledge synthesis projects in Medline or PubMed to exercise caution in using pure-MeSH filters, particularly the common filter ‘exp animals/not humans.sh’. In practical terms, the findings for human clinical RCTs suggest that this filter could remove one human study for every 100 automated-indexed records included in search results. This unintended removal of relevant evidence for knowledge synthesis projects is concerning. If possible, given result volumes and screening resources, we recommend against use of this filter until the automated indexing algorithm is improved. In particular, the filter should not be used for bodies of literature that are likely to use animal-related terminology – for example, studies of diets and dietary interventions.

The NLM has developed a new automated indexing system, termed MTIX, which is based on machine learning [6]. This system is asserted to significantly outperform MTIA in terms of accuracy: NLM testing found the F1 score (a combination assessment of recall and precision) for the human and animal check tags applied using MTIX to be 96% and 92% respectively [7]. In particular, NLM asserts that MTIX will be able to appropriately assess metaphorical language [7]. Future work will be necessary once this system has been fully implemented to assess whether it adequately addresses the problems of the MTIA system.

## LIMITATIONS

This study was conducted solely in Medline and assesses the impact of automated indexing specifically in the context of MeSH. As automated application of subject heading terms is extended to other databases [16], testing in those contexts will be required to assess whether those databases experience a similar rate of indexing concerns. Additionally, although automated indexing using MTIA is based solely on title-abstract and therefore a lack of abstract could significantly affect indexing [7], it was not possible to assess the impact of a lack of abstract on the results since the search sets had only a total of 16 studies without abstracts between them.

In evaluating whether particular records described a human study, we used a very broad definition of the term, including in vitro and ex vivo studies. A narrower definition may have impacted our findings. We also found that in some cases it was quite challenging to conclusively categorize studies as human or not, and we recognize that there may be some variation in analysis, particularly in two areas: use of human products on animal subjects, and agricultural/educational studies. We adopted a double-screen method for both title-abstract and full-text screening in an effort to provide a consensus-based confirmation of categorization.

While our study identifies human studies across several study types, the sets used for this analysis were developed using a search strategy specifically designed to retrieve randomized controlled trials. As such, further testing is needed to verify the impact on human-study filtering in other study types or contexts. Similarly, we identified some specific topics where our results suggest inappropriate exclusion of results may be more common, but the impact is likely to be variable across other search topics. Finally, our study was specific to use of the animals/humans check tags, but we believe there would likely be similar issues with other pure MeSH-based filtering approaches; additional research is warranted to confirm this suspicion and evaluate the extent of the problem.

As we conducted data extraction, we also noted that after our initial search, fourteen records had been changed from an indexing method of automated to curated, often because a human subject heading was added. While we view this as positive evidence of improvement in indexing by NLM, for the purposes of analysis we retained such studies in their original set. As noted by Amar-Zifkin and colleagues, these types of changes to indexing have a negative, though slight, impact on replicability [13]. As such, while retroactive reindexing could correct the errors of MTIA, it could also in itself cause problems for knowledge syntheses.

## Data Availability

Data associated with this article are available via the University of Manitoba Dataverse: https://borealisdata.ca/dataverse/AutomatedIndexing.

## SUPPLEMENTAL FILES

**Appendix A:** Medline search strategy

**Appendix B:** PRISMA flow diagrams
