# Supplementary material for: Filtering failure: the impact of automated indexing in Medline on retrieval of human studies for knowledge synthesis
Source: J Med Libr Assoc. 2025 Jan 14;113(1):58–64. doi: 10.5195/jmla.2025.1972 (PMC11835038; doi:10.5195/jmla.2025.1972)
Supplement: Supplementary file 1 — Appendix A: Medline search strategy [file jmla-113-1-58-s01.docx]

**Appendix A: Medline search strategy**

Ovid MEDLINE(R) and Epub Ahead of Print, In-Process, In-Data-Review & Other Non-Indexed Citations and Daily <1946 to March 10, 2023>

1 randomized controlled trial.pt. 588484

2 controlled clinical trial.pt. 95212

3 randomized.ab. 595485

4 placebo.ab. 236435

5 clinical trials as topic.sh. 200884

6 randomly.ab. 403681

7 trial.ti. 280912

8 1 or 2 or 3 or 4 or 5 or 6 or 7 1509064

9 exp animals/ not humans.sh. 5101357

10 8 not 9 1388660

11 8 not 10 120404

12 medline.st. 30287593

13 11 and 12 120404

14 limit 13 to yr="2021 - 2022" 10444

15 automated*.ig. 4218491

16 14 and 15 4865

17 curated.ig. 1196829

18 14 and 17 3062

19 14 not (16 or 18) 2517
