# Supplementary figures and images for: Filtering failure: the impact of automated indexing in Medline on retrieval of human studies for knowledge synthesis
Source: J Med Libr Assoc. 2025 Jan 14;113(1):58–64. doi: 10.5195/jmla.2025.1972 (PMC11835038; doi:10.5195/jmla.2025.1972)

**Appendix B: PRISMA flow diagrams**

***Automated indexing***

**
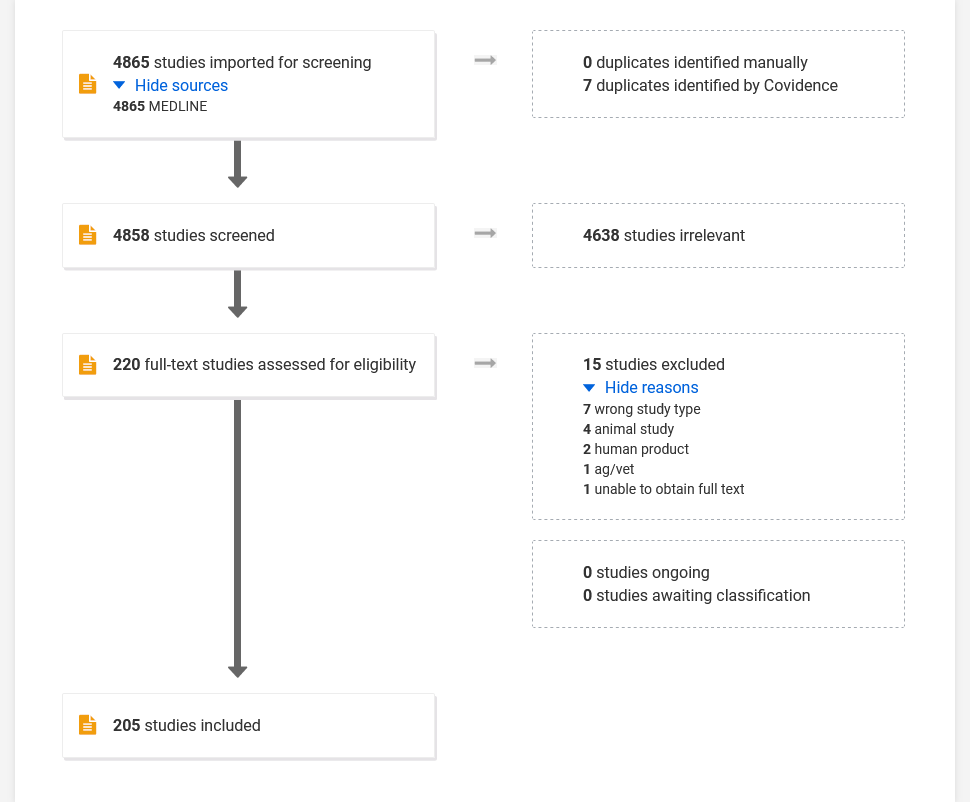
**

***Curated indexing***

**
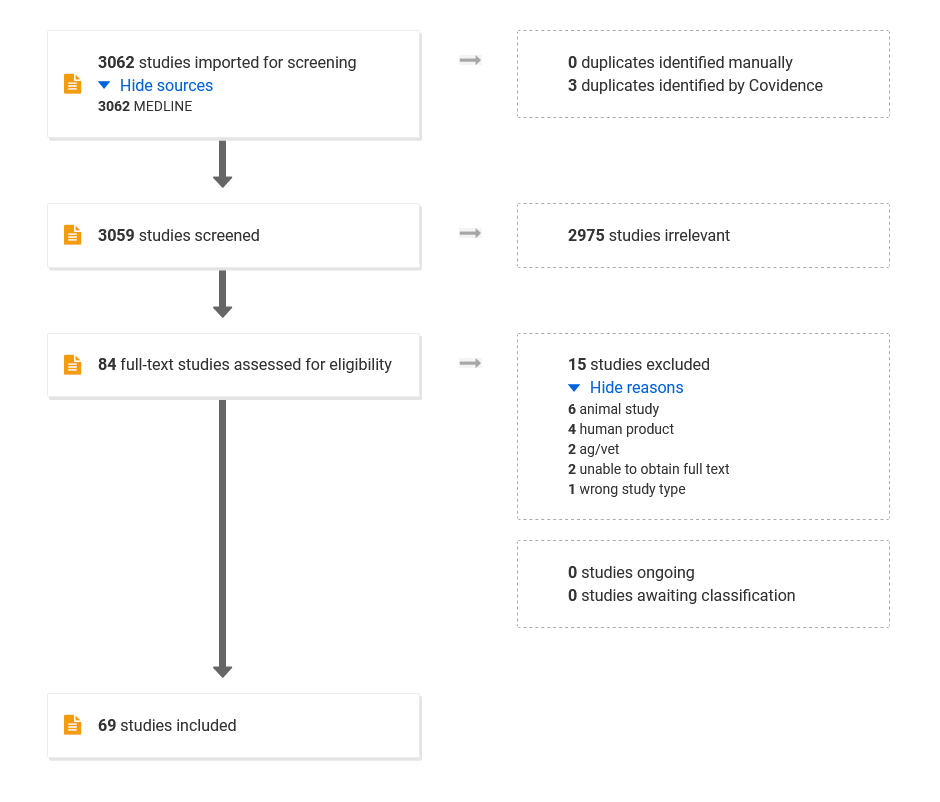
**

***Manual indexing***

**
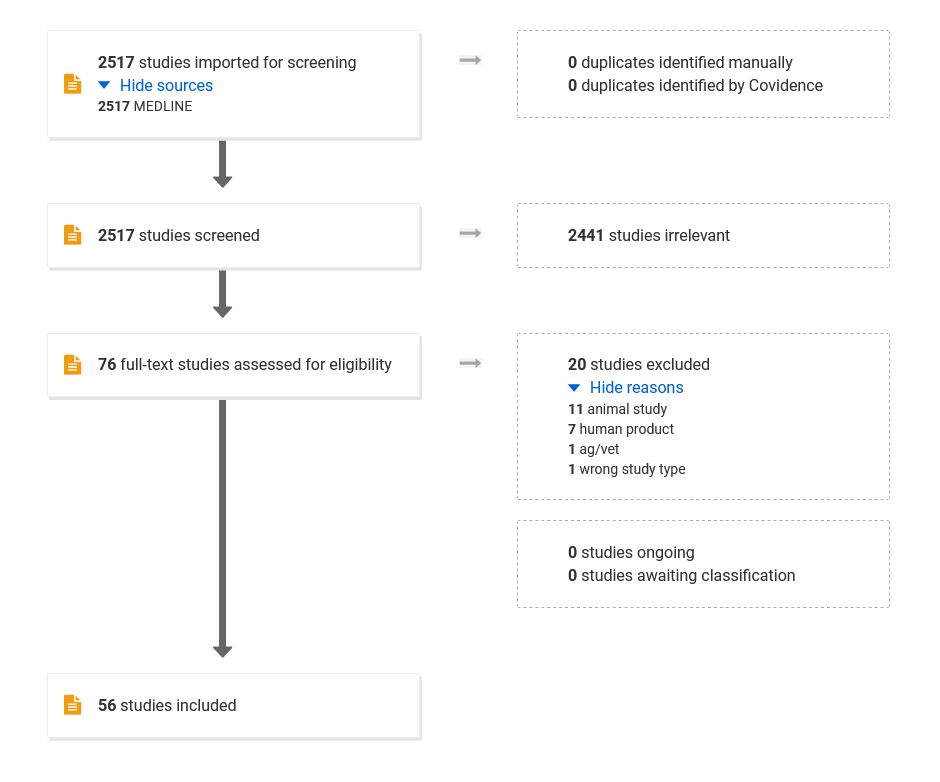
**

Supplement: Supplementary file 2 — Appendix B: PRISMA flow diagrams [file jmla-113-1-58-s02.docx]
